# Residential Traffic Exposure, Pulse Pressure, and C-reactive Protein: Consistency and Contrast among Exposure Characterization Methods

**DOI:** 10.1289/ehp.0901182

**Published:** 2010-02-02

**Authors:** Christine L. Rioux, Katherine L. Tucker, Mkaya Mwamburi, David M. Gute, Steven A. Cohen, Doug Brugge

**Affiliations:** 1 Department of Public Health and Community Medicine, Tufts University, Boston, Massachusetts, USA; 2 Department of Health Sciences, Northeastern University, Boston, Massachusetts, USA; 3 Department of Civil and Environmental Engineering, Tufts University, Medford, Massachusetts, USA

**Keywords:** C-reactive protein, inflammation, Puerto Rican, pulse pressure, residential traffic exposure, traffic analysis zone, traffic density, traffic proximity

## Abstract

**Background:**

Traffic exposure may increase cardiovascular disease (CVD) risk via systemic inflammation and elevated blood pressure, two important clinical markers for managing disease progression.

**Objectives:**

We assessed degree and consistency of association between traffic exposure indicators as predictors of C-reactive protein (CRP) and pulse pressure (PP) in an adult U.S. Puerto Rican population (*n* = 1,017).

**Methods:**

Cross-sectional information on health and demographics and blood data was collected. Using multiple linear regression, we tested for associations between CRP, PP, and six traffic exposure indicators including residential proximity to roads with > 20,000 vehicles/day and traffic density [vehicle miles traveled per square mile (VMT/mi^2^)]. Diabetes and obesity [body mass index (BMI) ≥ 30 kg/m^2^] were tested as effect modifiers.

**Results:**

CRP was positively associated with traffic density in the total population [36% CRP difference with 95% confidence interval (CI) 2.5–81%] for residence within the highest versus lowest VMT/mi^2^ level. With BMI ≥ 30, CRP showed significant positive associations with five of six traffic indices including residence ≤ 200 m versus > 200 m of a roadway [22.7% CRP difference (95% CI, 3.15–46.1)] and traffic density in the third highest versus lowest VMT/mi^2^ level [28.1% difference (95% CI, 1.0–62.6)]. PP was positively associated with residence within ≤ 100 m of a roadway for the total population [2.2 mmHg (95% CI, 0.13–4.3 mmHg)] and persons with BMI ≥ 30 [3.8 mmHg (95% CI, 0.88–6.8)]. Effect estimates approximately doubled for residence within ≤ 200 m of two or more roadways, particularly in persons with diabetes [8.1 mmHg (95% CI, 2.2–14.1)].

**Conclusions:**

Traffic exposure at roadway volumes as low as 20,000–40,000 vehicles/day may increase CVD risk through adverse effects on blood pressure and inflammation. Individuals with elevated inflammation profiles, that is, BMI ≥ 30, may be more susceptible to the effects of traffic exposure.

Results from clinical, epidemiologic, and animal studies together suggest that both short-term and long-term exposure to elevated levels of traffic have adverse effects on pulmonary and cardiovascular systems (Brook 2005; Brugge et al. 2007). Three interrelated biological pathways have been described to explain the mechanisms of action between inhaled pollutants, the lung, and the heart: *a*) disruption of the autonomic nervous system through irritant receptors and pulmonary nerve reflexes; *b*) stimulation of proinflammatory and pro-oxidative processes in the lung; and *c*) translocation of ultrafine particulates (UFP) (< 0.1 μm in aerodynamic diameter) components of air pollution directly into systemic circulation, eventually reaching the cardiovascular system (Brook 2008; Donaldson et al. 2005; Routledge and Ayres 2005). Disruption of the autonomic nervous system, inflammation, and oxidative stress are cross-promoting (Brook 2005), and disentangling this network of responses to traffic exposure has proved especially challenging in populations with preexisting conditions that exhibit complicated natural histories and treatment regimens.

Prior studies of C-reactive protein (CRP) have found associations with traffic-related exposures, although only one study focused on residential traffic exposure (Delfino et al. 2008). Pulse pressure (PP) has been reported in several studies as a strong predictor of adverse coronary outcomes and, for older populations, a stronger predictor than systolic blood pressure (SBP) (Blacher et al. 2000; Franklin et al. 2001; Haider et al. 2003; Zakopoulos et al. 2001). Several studies have examined associations between blood pressure and air pollution, although a limited number of these have examined PP (Auchincloss et al. 2008). To our knowledge, no prior study has evaluated the association of CRP, blood pressure measures, and residential traffic exposure using either direct measurements or other traffic indices at the local scale. The purpose of this study was to assess the impact of residential traffic exposure on systemic inflammation and PP in a cohort of older adults with relatively high prevalence of comorbidities associated with elevated inflammation. We hypothesized that individuals with conditions associated with elevated inflammation would have an increased inflammatory and blood pressure response to traffic stress. Further, we hypothesized that by assessing residential traffic exposure using several different traffic indices for local impacts near busy roads, as well as traffic density at the level of traffic analysis zones (TAZ), consistent and contrasting patterns of response would be seen, providing evidence for or against the alternate pathways between exposure and disease.

## Methods

### Study design and population

This cross-sectional analysis is part of a longitudinal cohort study on stress, nutrition, health, and aging conducted by the Boston Puerto Rican Center for Population Health and Health Disparities (CPHHD) at Tufts University. Health disparities previously documented in the Puerto Rican older adult population, including higher rates of diabetes and hypertension, prompted the larger study (Tucker 2005). Health status, demographic, blood, and genetic data were collected from 2004 to 2006 for 1,017 older Puerto Rican adults (45–75 years of age) recruited primarily through door-to-door enumeration (approximately 84%), with additional participants identified randomly during major citywide activities. Study design has been described elsewhere (Tucker 2005).

Because of the differing hypotheses under study, the selection criteria did not include traffic considerations. Participants resided primarily in the greater Boston, Massachusetts, area, with approximately 70% in the city of Boston. An additional 8–10% of participants resided in Chelsea and Lawrence, respectively, two areas of relatively high Puerto Rican density, with the remainder in surrounding cities and towns.

### Data collection

Interviews were collected in the homes of the participants by bilingual interviewers in either English or Spanish, as preferred by the participant. Blood samples were collected in the home by a certified phlebotomist on the day after the home interview. Blood pressure measurements were collected by a trained interviewer at three time points during the home visit, in duplicate. The second and third sets of readings, four readings in total, were averaged. Any readings found to be implausible or highly inconsistent were not included in the average. PP was calculated as the difference between the average SBP and diastolic blood pressure (DBP) values [see Supplemental Material (doi:10.1289/ehp.0901182) for additional details]. Anthropometric measurements were taken in duplicate, and the average of the two measures was used. Body mass index (BMI) was calculated as weight in kilograms divided by height in meters squared.

### Traffic exposure assessment

We used address geocoordinates to characterize residential traffic exposure at the time of the interview and acquisition of blood data. Residential traffic proximity was characterized for roadways with at least 20,000 vehicles/day, and multidirectional traffic density was characterized in terms of vehicle miles traveled per square mile (VMT/mi^2^) in each of 227 TAZs within the study area (Rioux et al. 2010).

We evaluated four indicators of roadway proximity: *a*) residential proximity ≤ 100 m of a roadway; *b*) residential proximity ≤ 200 m of a roadway ; *c*) number of roadways (0, 1, or ≥ 2) within 200 m of a residence; and *d*) a three-tiered exposure gradient (≤ 100 m, > 100 and ≤ 200 m, and > 200 m). The areas within 100 m or 200 m of either side of a roadway are referred to as 100-m and 200-m buffers.

Four traffic density exposure levels were defined, each representing an approximate 2-fold increase in VMT/mi^2^ across the study area, and study participants were assigned the traffic density level of the TAZ in which they resided [see Supplemental Material (doi:10.1289/ehp.0901182) for additional details]. A raster-based spatial density analysis was also conducted to examine the degree to which small TAZs may be influenced by the traffic levels of their contiguous TAZ. Density values represent a running weighted average of vehicle miles traveled within cells 10 × 10 m in diameter calculated over a 1,000-m radius from the center of each TAZ. Four raster-based exposure levels were defined, with level 4 representing the highest traffic density. Each level represents a 2-fold increase in raster-based VMT, and study participants were assigned the value for their residence (see Supplemental Materials for additional details).

Traffic data were obtained from the Central Transportation and Planning Staff of the Boston Region Metropolitan Planning Organization (MPO). A geographic information system–compatible file with traffic count station positions and measurement data collected by the Massachusetts Highway Department from 1997 to 2006 was used to identify approximately 60 roads of interest in the study area. Counts reflect traffic volumes in both directions.

### Data analysis

We evaluated the association between residential traffic exposure and both log-transformed levels of C-reactive protein (lnCRP) and PP by multivariate linear regression using SPSS software (version 16.0 for Windows; SPSS Inc., Chicago, IL). Results for lnCRP were back-transformed (exponentiated) to reflect the percent change, also called percent difference, in CRP for each unit increase in exposure. We use the term “percent difference” to avoid confusion, given the cross-sectional nature of this study.

We began a model-building process using stepwise regression as a screening process to evaluate the full set of explanatory variables presented in Table 1. These variables were chosen based on review of the literature and potential to contribute to preexisting inflammatory or hypertensive conditions or less than optimal management of such conditions. This process resulted in a slightly reduced set of variables used to adjust the separate outcome models for CRP and PP. Both outcomes were evaluated with respect to the full population as well as stratifications on the basis of BMI (< 30 and ≥ 30) and type 2 diabetes (glucose ≥ 126 mg/dL or taking diabetes medications) (Tucker et al. 2000). Both the CRP and PP models were adjusted for age, sex, BMI, waist circumference, glucose level, high-density lipoprotein (HDL), low-density lipoprotein (LDL), smoking status, diabetes, prior heart attack, heart disease, statin use, white blood cell (WBC) count, income/poverty ratio, and education. The CRP model also adjusted for the genetic variant rs1250 and albumin; the PP model was also adjusted for hypertension medication. Crude models were evaluated separately for each of the traffic variables and the two outcomes.

Each of the six traffic variables (four proximity and two density variables) were analyzed in separate models against the two outcomes, using the same set of outcome-specific covariates. Indicator dummy variables were used to evaluate the distance gradient (reference level > 200 m), the number of roadways near residence (zero as reference level), and the two traffic density approaches (the lowest density levels as reference levels).

## Results

### Study population and demographics

The mean age of study participants was 58 ± 7 years, and 72% were women (Table 2). The median length of time at their residence was 5 years, and 21% were employed. More than half were current or ex-smokers. Approximately 31% reported to be moderate drinkers (1–2 drinks/day) and 6.4% to be heavy drinkers (> 2 drinks/day). Only half had educational levels above the 9th grade.

The median CRP concentration of 3.7 mg/L was above the high-risk level for cardiovascular disease (Pearson et al. 2003). Age-based comparisons with the general population indicate that, except for older men, 75th percentile values for men (5.6 mg/L) and women (8.6 mg/L) ages 50–59 years and men (3.8 mg/L) and women (8.2 mg/L) ages 60–69 years in this study were generally higher than those reported for the National Health and Nutrition Examination Survey (NHANES), years 1999–2000 (Ford et al. 2004). Mean SBP and DBP were 136 ± 18.7 mmHg and 81 ± 10.7 mmHg, respectively, and a higher percentage of participants had systolic (38% > 140 mmHg) compared with diastolic (19% > 90 mmHg) hypertension. Mean PP was 54.9 ± 14.6. In a population with a mean age of 61 years, levels > 50 mmHg have been associated with adverse coronary outcomes (Haider et al. 2003).

### lnCRP, PP, and health status

CRP was significantly higher in participants with BMI ≥ 30 compared with BMI < 30, in those with versus those without type 2 diabetes, and in those who reported versus those who did not report heart disease (Table 2). These results are consistent with other studies (Fortmann 2004). PP was significantly higher in those with versus those without diabetes and in those who reported they had had a heart attack.

### Traffic exposure

Traffic volumes on most roads were between 20,000 and 40,000 vehicles/day, with only four roads between 40,000 and 100,000 vehicles/day and volumes on two roads > 100,000 vehicles/day. Less than 5% of participants classified as living near a road buffer lived near a road with between 40,000 and 100,000 vehicles/day, and< 3% lived near the highest-volume roads. Some individuals lived in locations both in proximity to a road and near areas of high traffic density; however, approximately 62% of individuals living in the highest two traffic density levels did not live within a 100-m buffer, and 37% did not live within a 200-m buffer (Table 3). Traffic density across the study ranged from 6,500 to 1,164,000 VMT/mi^2^, with a median of 88,000 VMT/mi^2^ [see Supplemental Material (doi:10.1289/ehp.0901182)].

Exposure distribution among those with health conditions was relatively equal with respect to traffic exposure except for BMI, with a significantly lower number (*p* = 0.031) of participants with BMI ≥ 30 living inside versus outside of a 100-m roadway buffer [see Supplemental Material (doi:10.1289/ehp.0901182)].

### Health outcomes: CRP

In the adjusted models, for the total population, a 36.2% difference [95% confidence interval (CI) 2.5–81] in CRP was associated with traffic density comparing the highest level (≥ 266,000 VMT/mi^2^) with the reference level (< 69,000 VMT/mi^2^) (Table 4). An 18.1% difference (95% CI, 1.3–37.6) was observed comparing the second-highest raster-based density level with the reference level. In stratified models, individuals with the highest levels of CRP (BMI ≥ 30) showed significant associations for five of six exposure characterization indices, and effect estimates were generally consistent with between 22.7 and 41.8 percent differences in CRP. When BMI was ≥ 30, a 22.7% difference (95% CI, 3.15–46) in CRP was associated with residing within ≤ 200 m compared with > 200 m of a roadway and a 28% difference (95% CI, 1.0–62.6) was associated with residing in the third highest versus lowest level of VMT/mi^2^.

The highest percent difference in CRP [71.3% (95% CI, 18.4–147)] was observed for individuals without diabetes residing within the highest versus lowest level of VMT/mi^2^. Changes in CRP were not significant for individuals with diabetes. Other variables that consistently remained significant for the CRP health outcome for the full population and stratified models include sex, BMI, waist circumference, glucose level, HDL, LDL, statin use, albumin, WBC count, and the CRP genetic variant. Crude models [see Supplemental Material (doi:10.1289/ehp.0901182)] were generally consistent with the adjusted models, with *p*-values in some cases being slightly lower in the crude models. A notable exception includes multiple roadway exposure for persons with BMI ≥ 30, where CRP was significant in the crude model (56% difference; 95% CI, 3.9–135) but not in the adjusted model.

**Figure 1:**
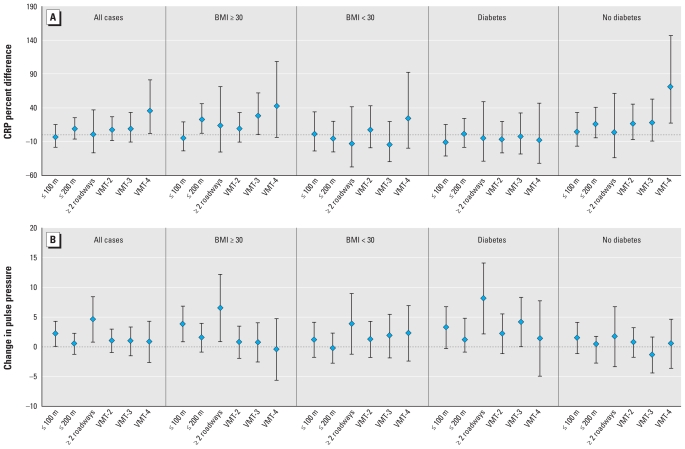
Percent difference in C-reactive protein (*A*) and difference in pulse pressure (*B*) and 95% CIs associated with traffic indices for complete study group and subgroups. Abbreviations: ≤ 100 m, location of residence ≤ 100 m of a roadway with more than 20,000 vehicles/day; ≤ 200 m, location of residence ≤ 200 m of these roadways; ≥ 2 buffers, location of residence within ≥ 2 200-m buffers of these roadways; VMT-2, VMT-3, VMT-4, traffic density levels associated with 69,000 to < 123,000 VMT/mi^2^, 123,000 to < 266,000 VMT/mi^2^, and ≥ 266,000 VMT/mi^2^, respectively.

Figure 1 presents the percent difference in CRP for all cases, individuals with BMI ≥ 30 and < 30, with and without type 2 diabetes for four exposure variables (≤ 100, ≤ 200, multiple roadways, and VMT/mi^2^).

### Pulse pressure

PP was positively associated with residence ≤ 100 m of a roadway with a difference of 2.2 mmHg (95% CI, 0.13–4.3 mmHg) for the total population and 3.8 mmHg (95% CI, 0.88–6.8) for those with BMI ≥ 30. Effect estimates were roughly double for residing ≤ 200 of two or more roadways, with a difference of 4.6 mmHg (95% CI, 0.81–8.4) for the total population and 6.5 mmHg (95% CI, 0.89–12.2) for persons with BMI ≥ 30. The highest effect estimates for PP [8.1 mmHg (95% CI, 2.2–14.1)] were observed for persons with diabetes residing within two or more roadways (Table 5). Other variables that consistently remained significant for the PP outcome for the full population and stratified models include age, BMI, waist circumference, glucose level, LDL, prior heart attack, heart disease, hypertension medications, and WBC count.

Figure 1 presents differences in PP for all cases, individuals with BMI ≥ 30 and < 30, diabetes and without diabetes for four exposure variables (≤ 100, ≤ 200, multiple roadways, and VMT/mi^2^).

## Discussion

CRP and PP are well-established risk factors for cardiovascular disease and are elevated among individuals with the conditions found to be prevalent in the Puerto Rican population. Health disparities have been previously documented in the Massachusetts Puerto Rican older adult population (Tucker 2005; Tucker et al. 2000) and include increased prevalence of diabetes even among nonobese individuals (Bermudez and Tucker 2001), poor therapeutic control of glycosolated hemoglobin, and higher prevalence of systolic hypertension compared with non-Hispanic whites (Lin et al. 2002). The inflammatory and hypertensive profile seen in this study population, although complicated by the use of medications such as statins, insulin, oral hypoglycemic medications, and antihypertensive medications, may provide insights into inherent and acquired susceptibilities as well as mediating factors. The health disparities reported in this population may indicate a higher level of vulnerability to traffic-related health effects. Our findings are most generalizable to other age-matched, residence- and income-matched populations with similar health profiles, namely, elevated BMI.

Differential personal exposure to particles, gaseous pollutants, and traffic pollution have been associated with lower socioeconomic position with respect to education, minority status, and income and major roadways have been routed through lower-income areas with less political and economic power (O’Neill et al. 2003). Here we controlled for income/poverty ratio and educational level in all models. We also examined as covariates working status and length of time at residence, although neither was observed to be significant, or even close to significant, in the model-building stage. Only 18.5% of this study population reported to be working. This is the first study of which we are aware that examines traffic-related health effects in the older Puerto Rican population.

### Overview of health outcomes

Individuals with higher CRP, that is, individuals with BMI ≥ 30, had significant associations with traffic exposure consistent with inflammation mediating the effects of traffic exposure. Inflammatory processes in the airways and lungs can prompt signaling in autonomic fibers (Utell et al. 2002), suggesting the two pathways interact. Inflammation (Hansson 2005; Libby and Ridker 2004) and PP (Dart and Kingwell 2001) play central roles in the development and progression of atherosclerosis. There is evidence that PP, acting through the promotion of endothelial damage and mechanical fatigue, may be both a cause and consequence of atherosclerosis, which in turn promotes aortic stiffness and increased central wave reflection associated with elevated PP (Dart and Kingwell 2001).

We also examined SBP and DBP, WBC counts, and percent neutrophils, which are the most numerous WBCs, as outcomes (detail not shown). In persons with BMI ≥ 30 only, SBP was positively associated (*p* = 0.013) with proximity ≤ 100 m [4.5 mmHg (95% CI, 0.46–8.6)], confirmed by the distance gradient (*p* = 0.017) variable ≤ 100 m [4.8 (95% CI, 0.87–8.76)]. SBP was also positively associated (*p* = 0.045) with traffic density in the third versus reference level for persons with diabetes [5.6 (95% CI, 0.12–11)]. Associations remained significant (*p* < 0.05) after removing the two highest outliers for SBP (> 200 mmHg).

For persons with BMI ≥ 30, DBP was negatively associated (*p* = 0.013) with residence near multiple roadways [−5.4 (95% CI, −9.6 to −1.1)] and remained significant after removing the two lowest outliers for DBP (< 53 mmHg). These findings may partially explain the significant increases in PP observed mostly for proximity ≤ 100 m and multiple roadways, although the associations in PP are somewhat more consistent for the full population, persons with BMI ≥ 30, and persons with diabetes. In the full population in adjusted models, associations remained significant (*p* < 0.05) for PP and proximity ≤ 100 m and multiple roadways after removing the two lowest outliers for DBP (< 53 mmHg). Associations also remained significant (*p* < 0.05) for PP and multiple roadways in the full population after removing the two highest outliers for SBP (> 200 mmHg) but became borderline significant (*p* = 0.059) for proximity ≤ 100 m. Associations for PP did not change after removing DBP and SBP outliers for persons with BMI ≥ 30.

In persons with BMI ≥ 30 only WBC counts were positively associated [0.59 × 10^3^/mm^3^ (95% CI, 0.015–1.173)] with the distance gradient ≤ 100 m compared with > 100 to ≤ 200 m and > 200 m. The association was not confirmed in the proximity ≤ 100 variable. No significant associations were observed for neutrophils.

### Overview of traffic indices

Increased traffic density and/or proximity to major roadways have been associated with several adverse health outcomes related to the heart and lung (Gauderman et al. 2005; Hoffmann et al. 2007, 2009; Kim et al. 2008; Lipfert et al. 2006; McConnell et al. 2006; Medina-Ramón et al. 2008; Tonne et al. 2007). Traffic density has been identified as a significant predictor of nitrogen oxides (NO_x_), nitrogen dioxide (NO_2_), particulates < 2.5 μm in aerodynamic diameter (PM_2.5_), the soot content of PM_2.5_, and volatile organic chemicals in a review of 25 studies using land use regression analysis to characterize traffic exposure (Hoek et al. 2008). Pollution gradients reported in the vicinity of highways in the United States with over 100,000 vehicles/day show that concentrations of ultrafine particles, carbon monoxide (CO), black carbon, and NO_x_ drop off exponentially with distance (Zhang et al. 2004; Zhu et al. 2002a, 2002b). Steep declines in UFP concentrations have also been reported for a lower-volume road (30,000 vehicles/day) in England (Shi et al. 1999).

We used four methods to assess traffic proximity, and two methods were used for traffic density. In addition to the 100-m and 200-m roadway buffers, a distance gradient was developed to test for the presence of a dose–response relationship. Assessment of multiple roadway exposure could indicate whether impacts were additive or multiplicative. The traffic density variable VMT/mi^2^ assessed multidirectional effects common in dense urban areas and tested the utility of TAZ data for this purpose. The raster-based spatial density assessment examined the degree to which small-area TAZs may be influenced by the traffic levels of contiguous TAZs.

For roadway proximity indices in adjusted models, PP was significant for the total population, individuals with BMI ≥ 30, and with diabetes, whereas CRP was significant for individuals with BMI ≥ 30. In crude models, CRP was also significant for the full population and for individuals without diabetes. Roadway proximity ≤ 100 m was significant for PP, whereas proximity ≤ 200 m was significant for CRP. A disproportionately lower number of individuals with BMI ≥ 30 lived within versus outside of a 100-m roadway buffer, possibly explaining the lack of association for CRP at this distance. PP may be a stronger predictor of traffic proximity among this group. An alternate explanation would be the different composition of traffic-related air pollution (TRAP) within the two gradients. For example, primary emissions comprising largely fresh UFP closest to highways differ in chemical composition from mature UFP formed downgradient from the vehicular emission source (Brugge et al. 2007). Higher noise levels closer to roadways may be a greater factor for PP versus CRP, although evidence associating noise with individual measures of SBP and DBP is inconsistent (Babisch 2006).

Residing near multiple roadways was consistently associated with PP, with effect estimates approximately double those for the proximity variable suggesting additive effects. Of the 218 cases residing ≤ 100 m of a roadway, 74% (*n* = 162) lived near one roadway and 26% (*n* = 56) resided near two or more roadways.

The distance gradient compared residence within a certain roadway distance (≤ 100 m or > 100 to ≤ 200 m) with the reference of > 200 m. These indices were always consistent with the proximity indices indicating that ≤ 100 m was the higher impact zone for PP, but for CRP the > 100 to ≤ 200-m zone was more relevant, specifically for individuals with BMI ≥ 30. Aside from the different pollutant and noise gradients, building type and design closer to roadways and level of activity and time spent at home could also be factors contributing to a difference in exposure and response.

The two density methods, TAZ-based and raster-based, were consistent with each other although the smoothing function in the raster-based density tended to eliminate some of the exposure contrast, with more cases shifted into lower raster-based density levels compared with the higher TAZ-based levels. For three population classes [all cases, BMI ≥ 30 and without diabetes (crude model)] where raster-based density had a significant association with CRP, the associations were seen in the second density level (level 2) compared with the reference level (level 1), whereas with TAZ-based density, significant associations were seen in the highest density level. The difference in outcomes associated with proximity and density may be partially explained by *a*) the distribution of individuals who lived in high-traffic density areas but did not live within a 100-m or 200-m buffer; and *b*) the general underrepresentation of cases in the high-traffic areas—for example, 60 of 923 cases resided in the highest traffic density level.

The lower level of traffic in terms of roadway volumes or traffic density associated with adverse health effects has not been established. Studies have used different definitions of what constitutes a major roadway. Several studies have reported adverse health effects associated with residing or attending school near interstates, highways, and major arterials (McConnell et al. 2006; Van Hee et al. 2009) or for major roads with vehicle counts that ranged from 10,000 to 100,000 (Hoffmann et al. 2006, 2007, 2009). Major arterials can range from 15,000 to 30,000 vehicles/day. These studies did not report the number or percentage of subjects living near the lower versus higher range of traffic volumes. A lower volume threshold common to many of the surface roads throughout the study area was evaluated in this study, and results strongly suggest residence near roadways with traffic volumes between 20,000 and 40,000 vehicles/day may be associated with adverse outcomes. More than 90% of study participants resided near roads between 20,000 and 40,000 vehicles/day. Separate analyses were conducted excluding those participants residing near the highest-volume roads, and results were unchanged (not shown).

### Consistency with studies of CRP

Some prior studies of CRP found associations with traffic-related exposures consistent with the findings reported here, although only one study focused on residential traffic exposure. In a study of 29 nonsmoking elderly people with a history of coronary artery disease, significant associations were reported between several markers of inflammation including CRP and indoor and outdoor concentrations of several traffic-related pollutants including PM_2.5_, elemental carbon, black carbon, particle number, CO, and NO_x_ (Delfino et al. 2008). An association with CRP above the 90th percentile was reported with ultrafine traffic-related particles measured at central monitors approximately 1 km away from a clinic where blood samples were collected (Yue et al. 2007). Rückerl et al. (2006) evaluated several pollutant classes related to traffic, including concentrations of elemental and organic carbon, ultrafine particles from 0.01 to 0.1 μm, and accumulation mode particle counts for particles from 0.1 to 1.0 μm collected from a central monitor. Significant associations with CRP above the 90th percentile in individuals attending a clinic were reported for ultrafine and accumulation mode particle fractions. Another study by the same authors found no consistent associations with CRP and several traffic-related pollutants (Rückerl et al. 2007).

Measuring PM_2.5_ in highway patrol cars, Riediker et al. (2004) reported significant associations with several inflammatory biomarkers including CRP. Using a combination of central monitoring and personal microenvironment monitoring data for PM_2.5_, a significant association was reported between CRP and ambient levels, particularly among persons with diabetes, obesity, and hypertension, including combinations of these conditions (Dubowsky et al. 2006). One study used central monitors combined with air dispersion modeling to predict levels of PM_10_ (particulates < 10 μm in aerodynamic diameter), NO_2_, sulfur dioxide (SO_2_), and ozone at the postal code level closest to residence but found no association with CRP (Forbes et al. 2009). Our results are generally consistent with studies reporting associations with CRP above the 90th percentile. For those with preexisting proinflammatory conditions as in this study, individuals with BMI ≥ 30 had the highest CRP levels compared with the other subgroups.

### Consistency with studies of PP

Several studies have examined associations between blood pressure measures and air pollution, some of which examined air pollution specifically related to TRAP. Only one study addressed PP and found significant associations with PP and SBP and PM_2.5_ based on central air monitoring data (Auchincloss et al. 2008). Distance to roadway and traffic density measured as roadway length around a residence increased these associations but were not independently associated with PP and SBP. A three-city study examined acute exposure to UFP, accumulation mode particles, and PM_2.5_ at central air pollution monitors located in each city. Small but significant decreases with SBP and DBP were reported for all lag times from 0 to 5 days (Ibald-Mulli et al. 2004). In a controlled experiment of 23 healthy young adults (average age 23 ± 10 years), the magnitude of change in DBP was associated with the organic carbon content of PM_2.5_, suggesting a traffic-related mechanism (Urch et al. 2005). Significant changes in SBP were not observed. In a study not specifically targeted to traffic exposure, small but statistically significant differences in SBP (1.79 mmHg per 90 μg/m^3^ total suspended particulates and 0.74 mmHg per 80 μg/m^3^ SO_2_) were reported for 2,607 men and women 25–64 years of age (Ibald-Mulli et al. 2004). Cardiac rehabilitation patients showed positive associations with SBP, DBP, and mean arterial pressure and PM_2.5_ measured from central monitors (Zanobetti et al. 2004). A recent study designed to test the association between air pollution and blood pressure, systemic inflammation, and endothelial dysfunction reported significant increases in DBP and decreases in heart rate variability immediately following a controlled 2-hr exposure to concentrated PM_2.5_ in an area reported to be heavily influenced by traffic (downtown Toronto, ON, Canada) (Brook et al. 2009). Because of differences in response time, the authors conclude that the immediate-onset increases in blood pressure were a result of acute autonomic nervous system imbalance and not attributable to increases in inflammatory markers or observed increases in flow-mediated dilation that decreased significantly after 24 hr. Later-onset changes in blood pressure may be attributable to these mechanisms (Brook et al. 2009).

Two components of traffic exposure are relevant to blood pressure measures: traffic-related air pollution and roadway noise. A review of > 60 studies on transportation noise and cardiovascular risk concluded there was sufficient evidence for an association between traffic noise and ischemic heart disease, limited to sufficient evidence for an association with hypertension, and no consistent evidence for the individual SBP and DBP measures (Babisch 2006). None of the studies were reported to have specifically addressed PP. The author noted that several issues may dilute effect, including migration of sensitive people out of high-noise areas, medication, and the exclusion of subjects with preexisting conditions or hypertension when individual measures of blood pressure were studied. Despite inconsistent results, the increase in noise levels with traffic volumes, and decreasing noise levels with distance (6 dB per doubling of distance from the road) have been well established (Ouis 2001). Based on available data, the contribution of traffic noise to elevated PP cannot be excluded in this study.

### Negative associations

Contrary to a prior study, we found that those without, rather than with, diabetes had higher significant associations with traffic for some traffic indices, including traffic density, and to a lesser extent with proximity within 200 m of a road (Dubowsky et al. 2006). Although not statistically significant, individuals with diabetes showed a consistent negative association with CRP and almost all traffic indices in what could be a result of the anti-inflammatory effects of noninsulin medications (Chu et al. 2002; Dandona et al. 2004).

A consistently negative association, although not significant, was also observed between CRP and all traffic indices for individuals with BMI < 30. Interleukin 6 (IL-6), the cytokine primarily responsible for stimulation of CRP, is produced in adipose tissue, and both IL-6 and CRP are found at higher circulating levels in individuals with higher body fat (Dandona et al. 2004; Yudkin et al. 1999, 2000). The negative associations seen in nonobese individuals could also be the result of an anti-inflammatory effect of diabetes medications in a population with generally lower adipose-generated levels of IL-6 and lower CRP. Approximately 30% of those with BMI < 30 have diabetes, although the level of diabetes among those with BMI ≥ 30 is > 50%. The use of statins, also shown to have anti-inflammatory effects (Albert et al. 2001; Ridker et al. 1999) is approximately 30% in those with BMI < 30 and 42% in those with BMI ≥ 30. The role of anti-inflammatory medications in mediating the inflammatory response to traffic will be examined in a future paper (Rioux CL, Tucker KL, Brugge D, Gute DM, Mwamburi M, unpublished data).

### Strength and limitations

Our findings may be limited in their generalizability. The study population was Puerto Rican, predominantly low income, and had high prevalence of preexisting cardiovascular disease. It is probably reasonable, however, that our findings be compared with other older populations with high degrees of morbidity. Although this analysis provides new evidence regarding traffic-related health impacts at the local scale, the cross-sectional nature of the study and the use of surrogate rather than direct measures of traffic-related noise and air pollution are limitations. Surrogates of traffic exposures such as road proximity and traffic density, although capturing some of the variation in local traffic environments, do not capture the actual temporal and spatial variations or migration and degradation profiles of the actual pollutant mix that originates from roadways that may be the underlying causal agents for adverse health effects. Time and activity relationships among the exposed population, including the relative time spent inside and outside of the home and differences between ambient air concentrations and those in the indoor environment that originate from outdoor sources, are important considerations that we did not account for. The degree to which traffic-related health effects result from acute versus chronic exposures remains an ongoing research area. A combination of daily peak or shorter-term exposures and longer-term exposures is likely to contribute to the underlying mechanisms for cardiovascular illnesses. The biomarkers we examined, PP and CRP, have been associated with both short-term and longer-term exposures. The median length of residence in our study population was 5 years, and no consistent difference in outcomes was observed when models were stratified by length of residence. For outcomes like lung cancer, longer exposure durations may be more relevant; however, we remain convinced that residential exposures in the time frames observed for this study population are meaningful for the biomarkers examined.

The TAZ approach, despite limitations, was a robust predictor of CRP, which was generally confirmed by other traffic indices within a subpopulation group, for example, individuals with BMI ≥ 30. Notable among the limitations of the TAZ approach was the potential for exposure misclassification for individuals residing near a TAZ boundary but who would be expected to be influenced to some degree by the traffic impacts of an adjacent TAZ. The raster-based density approach was developed to address these intra-TAZ influences. Another limitation of the TAZ approach is that the traffic density estimates encompass automobiles and trucks but not buses, which may be a significant contributor to the overall pollution load in certain areas (Levy et al. 2001).

People residing within the 200-m buffers may have very different exposure levels as a result of their exact distance from the road, the location of apartments within a building, the condition of windows, infiltration rates, and ventilation systems within the building. Ambient levels of traffic-related pollution and noise are higher at locations within meters of a roadway than at the farthest edge of the 200-m buffer. Topographic features such as trees and other buildings may also reduce traffic impacts within the 200-m buffer. A review of wind direction distribution from Boston Logan Airport in Boston indicates a changing pattern of wind directions in the Boson area. Five months of the year, the predominant wind direction is from the west (fall and winter), 6 months from the east (spring and summer), and 1 month (July) from the south (Windfinder 2009). Orientation of homes with respect to roadways was not considered and could influence the magnitude of exposure, with increasing exposures downwind versus crosswind. People spend different amounts of time at home, reflecting variation in actual residential traffic exposure. A low employment rate (20%) among this older population suggests time spent at home was relatively high, although variation could arise from travel outside the home for purposes other than employment.

Strengths of this study include the extent of covariate information on health-related behaviors (smoking, alcohol consumption, physical activity levels), comorbidities (hypertension, prior heart attack, and heart disease), physiologic markers of health status (HDL, LDL, blood pressure, and other markers of inflammation including serum albumin, platelet, and WBC counts), and socioeconomic factors (income/poverty levels, education, health insurance, and other social support factors) that could be used to develop and adjust the models and develop a clearer picture of the associations between traffic and the outcome of concern. Traffic data, including traffic count statistics on vehicles per day and the TAZ-level traffic density information, allowed for a detailed characterization of the variability of traffic exposure conditions across the study area.

## Conclusions

In this study we found that adverse health effects were associated with residence near roadways with traffic volumes between 20,000 and 40,000 vehicles/day, suggesting risks from residential exposure at lesser roadway volumes than previously reported. Individuals with BMI ≥ 30 had higher levels of CRP and exhibited stronger statistical associations with traffic exposure indices and both CRP and PP. These findings provide suggestive evidence that *a*) inflammation may mediate the effects of traffic exposure; and *b*) individuals with conditions associated with elevated inflammatory profiles may be particularly susceptible to the adverse effects of traffic exposure. Assessment of residential traffic exposure using several different traffic indicators is a robust method for investigating the role of distance to roadway, traffic volume, dose response, cumulative impacts, and high-volume, single-source versus multidirectional overall traffic density on adverse health outcomes from traffic.

## Figures and Tables

**Table 1:** Baseline characteristics for the 1,017 Boston Puerto Rican Center for Health and Health Disparities Study participants, Greater Boston, Massachusetts, USA.

Characteristic	Total sample (*n* = 1,017)	≤ 200 m (*n* = 432)	> 200 m (*n* = 585)	*p*-Value
Basic descriptive				
Age (years)	57.8 ± 7.4	57	57	0.464
Female sex	731 (72)	316	415	0.439
Income/poverty ratio < 100	573 (59)	250	323	0.371
Employment	189 (21)	63	126	0.007

Smoking status				0.06

Smoker	235 (23.3)	196	273	
Ex-smoker	303 (30.1)	118	185	
Never smoker	469 (46.6)	115	120	

Alcohol consumption				0.946

Not current	626 (62.5)	263	362	
Current moderate	311 (31.1)	134	177	
Current heavy	64 (6.4)	28	36	

Educational level				0.07

< 5th grade	237 (23.3)	103	134	
5th–9th grade	263 (25.9)	123	140	
9th–12th	360 (35.5)	154	205	
Some college	132 (13)	47	86	
Some graduate school	23 (2.3)	5	18	

Health status				
BMI (kg/m^2^)	32 ± 6.8	31.9	32.3	0.467
Waist circumference (cm)	102 ± 15.2	1.02	1.02	0.795
Glucose level (mg/dL)	123 ± 54	120	125	0.142
HDL (mg/dL)	44.9 ± 12.6	44.2	45.6	0.116
LDL (mg/dL)	107 ± 34.9	105	109	0.104
SBP (mmHg)	136 ± 18.7	137	135	0.276
DBP (mmHg)	81.1 ± 10.7	80.1	81.3	0.36
PP (mmHg)	54.9 ± 14.6	56.1	54.2	0.04
Hypertension	709 (70.3)	307	402	0.383
Heart attack (self-reported)	94 (9.3)	43	51	0.494
Heart disease (self-reported)	135 (13.3)	54	81	0.541

Medications				
Statins	381 (37.5)	169	212	0.348
Hypertension	563 (55.6)	247	316	0.319
Insulin	108 (10.6)	47	60	0.749
Oral hypoglycemic agents	232 (22.7)	100	132	0.826
Nonsteroidal anti-inflammatory drugs	353 (37.5)	146	207	0.599

Confirmed conditions				
Type 2 diabetes	417 (41.7)	180	237	0.579
Obesity (BMI ≥ 30)	586 (58.3)	236	350	0.093

Inflammatory biomarkers				
CRP (mg/L)	3.7 (0–127)	6.5	5.9	0.246
Log-transformed CRP (mg/L)	1.19 ± 1.18	1.24	1.16	0.282
Serum albumin (g/dL)	4.3 ± 0.32	4.24	4.28	0.07
WBC (10^3^ cells/μL)	6.9 ± 2.4	7.1	6.7	0.014

Values are *n* (%), mean ± SD, when normally distributed, and median (range) when other distribution. *p*-Values are derived from Pearson chi-square for binary variables and *t*-test for independent samples for continuous variables.

**Table 2:** Ln-transformed C-reactive protein concentrations and pulse pressure levels by health status.

Outcome	BMI < 30	BMI ≥ 30	*p*-Value	No type 2 diabetes	Type 2 diabetes	*p*-Value	No prior heart attack	Prior heart attack	*p*-Value	No heart disease	Heart disease	*p*-Value
lnCRP (mg/L)	*n* = 404	*n* = 565		*n* = 578	*n* = 403		*n* = 890	*n* = 90		*n* = 850	*n* = 130	
	0.68 ± 1.14	1.54 ± 1.05	0.001	1.07 ± 1.18	1.36 ± 1.15	< 0.001	1.19 ± 1.18	1.12 ± 1.11	0.545	1.13 ± 1.17	1.53 ± 1.11	< 0.001
Pulse pressure (mmHg)	*n* = 415	*n* = 562		*n* = 572	*n* = 400		*n* = 896	*n* = 92		*n* = 858	*n* = 130	
	54.7 ± 14.4	55.1 ± 14.8	0.627	52.5 ± 13.8	58.7 ± 15.2	< 0.001	54.2 ± 14.3	62.3 ± 16.3	< 0.001	54.9 ± 14.7	56 ± 14.5	0.395

Values are mean ± SD. Case numbers vary by outcome based on exclusion criteria of WBC counts for lnCRP. *p*-Values are based on independent samples *t*-test.

**Table 3:** Number (%) of participants residing within traffic proximity categories (≤ 100 m and ≤ 200 m of roadways) and traffic density levels (VMT/mi^2^), Greater Boston, Massachusetts, USA.

	Traffic density levels (VMT/mi^2^)				
Proximity measure	Level 1 < 69,000	Level 2 69,000 to < 123,000	Level 3 123,000 to < 266,000	Level 4 ≥ 266,000	Total
≤ 100 m					
No	334 (88)	302 (81)	123 (62)	39 (57)	798
Yes	47 (12)	69 (19)	74 (38)	28 (41)	218
Total	381	371	197	68	1,017

≤ 200 m					
No	275 (72)	213 (57)	79 (40)	18 (26)	585
Yes	106 (28)	158 (43)	118 (60)	50 (74)	432
Total	381	371	197	68	1,017

**Table 4:** Percent difference (95% CI) in C-reactive protein associated with traffic indices for the complete study group and subgroups.^a^

	All cases		Obesity				Diabetes			
Traffic index		*p*-Value	Yes	*p*-Value	No	*p*-Value	Yes	*p*-Value	No	*p*-Value
≤ 100 m	−2.7 (−18.0 to 15.4)	0.747	−4.3 (−23.2 to 19.2)	0.697	1.3 (−23.4 to 33.9)	0.927	−10.6 (−30.9 to 15.7)	0.393	4.6 (−16.2 to 33.1)	0.645
≤ 200 m	8.9 (−5.3 to 25.4)	0.229	22.7 (3.15 to 46.1)	0.021	−5.1 (−24.9 to 20.2)	0.668	1.5 (−17.6 to 25.2)	0.885	16.1 (−4.3 to 40.6)	0.129
3-Tiered gradient										
≤ 100 m	1.4 (−15.0 to 20.9)	0.878	4.6 (−16.5 to 30.9)	0.697	−1.3 (−26.1 to 32)	0.932	−7.9 (−29.5 to 20.2)	0.540	10.9 (−12.6 to 41.1)	0.393
> 100 to ≤ 200 m	17.1 (−1.8 to 39.6)	0.079	41.8 (14.2 to 75.9)	0.002	−8.9 (−32.6 to 22.9)	0.538	11.5 (−14.2 to 44.8)	0.414	21.7 (−4.8 to 55.6)	0.117
No. of roadways										
1 roadway	10.3 (−4.7 to 27.6)	0.188	23.9 (3.5 to 48.6)	0.02	−3.7 (−24.7 to 23.1)	0.761	2.5 (−17.7 to 27.9)	0.822	17.8 (−3.6 to 43.9)	0.109
≥ 2 roadways	0.5 (−26.3 to 37.2)	0.973	13.7 (−24.4 to 71.3)	0.535	−12.9 (−46.6 to 42)	0.580	−4.1 (−38.4 to 49.3)	0.853	4.2 (−32.8 to 61.8)	0.853
VMT/mi^2^										
Level 2	8.0 (−8.1 to 26.9)	0.349	9.6 (−9.9 to 33.5)	0.359	7.8 (−18.7 to 43)	0.600	−5.7 (−25.9 to 20.2)	0.634	16.5 (−6.6 to 45.2)	0.175
Level 3	9.5 (−9.9 to 33.1)	0.360	28.1 (1.0 to 62.6)	0.041	−14.5 (−39.3 to 20.4)	0.369	−2.4 (−27.9 to 32.2)	0.876	18.3 (−8.8 to 53.4)	0.204
Level 4	36.2 (2.5 to 81.1)	0.033	42.5 (−3.1 to 109)	0.072	24.5 (−19.7 to 92.9)	0.326	−7.4 (−41.6 to 46.8)	0.742	71.3 (18.4 to 147)	0.004
Raster density										
Level 2	18.1 (1.3 to 37.6)	0.034	25.9 (4.3 to 51.8)	0.016	8.2 (−16.9 to 40.8)	0.557	17.7 (−6.7 to 48.6)	0.169	20.6 (−2.1 to 48.6)	0.078
Level 3	13.2 (−9.1 to 40.9)	0.267	8.0 (−18.8 to 43.8)	0.595	18.6 (−16.9 to 69.6)	0.344	1.2 (−27.5 to 41.3)	0.942	23.1 (−8.5 to 65.5)	0.169
Level 4	6.6 (−29.3 to 60.6)	0.761	23.0 (−23.5 to 97.6)	0.392	−32.8 (−69.4 to 47.8)	0.322	42.9 (−22.12 to 162)	0.249	−10.7 (−49.4 to 57.8)	0.696

Reference levels were > 100 m for ≤ 100 m; > 200 m for ≤ 200 m and the 3-tiered gradient; 0 (roadways) for number of roadways ≤ 200 m of a residence; and level 1 for VMT/mi^2^ and raster density.

a All CRP models were adjusted for age, sex, BMI, waist circumference, glucose level, HDL, LDL, smoking status, diabetes, prior heart attack, heart disease, statin use, albumin, WBC count, CRP genetic variant rs1250, income/poverty ratio, and education.

**Table 5:** Difference in pulse pressure (95% CI) associated with all traffic indices for the complete study group and subgroups.^a^

	All cases		Obesity				Diabetes			
Traffic index		*p*-Value	Yes	*p*-Value	No	*p*-Value	Yes	*p*-Value	No	*p*-Value
≤ 100 m	2.2 (0.13 to 4.3)	0.038	3.8 (0.88 to 6.8)	0.011	1.2 (−1.8 to 4.1)	0.432	3.2 (−0.24 to 6.8)	0.068	1.5 (−1.1 to 4.1)	0.152
≤ 200 m	0.6 (−1.2 to 2.3)	0.524	1.6 (−0.8 to 3.9)	0.198	−0.19 (−2.7 to 2.3)	0.881	1.2 (−0.89 to 4.9)	0.176	0.46 (−2.6 to 1.7)	0.681
3-Tiered gradient										
≤ 100 m	2.0 (0.04 to 3.9)	0.073	3.7 (0.68 to 6.8)	0.017	0.8 (−2.3 to 3.9)	0.605	3.4 (−0.23 to 7.0)	0.066	1.0 (−1.7 to 3.7)	0.468
> 100 to ≤ 200 m	−0.9 (−3.1 to 0.9)	0.422	−0.4 (−3.3 to 2.5)	0.789	−1.4 (−4.7 to 1.8)	0.402	−0.56 (−3.1 to 5.2)	0.762	−2.1 (−4.8 to 0.74)	0.149
No. of roadways										
1 roadway	−0.045 (−1.8 to 1.8)	0.961	0.9 (−1.5 to 3.4)	0.465	−0.88 (−3.5 to 1.7)	0.507	0.9 (−2.1 to 3.9)	0.504	−0.71 (−3.0 to 1.5)	0.516
≥ 2 roadways	4.6 (0.81 to 8.4)	0.018	6.5 (0.89 to 12.2)	0.024	3.8 (−1.2 to 8.9)	0.134	8.1 (2.2 to 14.1)	0.007	1.7 (−3.3 to 6.7)	0.497
VMT/mi^2^										
Level 2	1.0 (−0.94 to 3.0)	0.302	0.80 (−1.9 to 3.5)	0.559	1.3 (−1.5 to 4.1)	0.404	2.2 (−1.1 to 5.5)	0.188	0.8 (−1.8 to 3.3)	0.553
Level 3	0.98 (−1.4 to 3.4)	0.424	0.78 (−2.5 to 4.0)	0.644	1.9 (−1.8 to 5.4)	0.312	4.1 (−0.01 to 8.3)	0.051	−1.3 (−4.3 to 1.7)	0.383
Level 4	0.87 (−2.6 to 4.3)	0.621	−0.4 (−5.6 to 4.8)	0.876	2.3 (−2.3 to 6.9)	0.328	1.4 (−4.9 to 7.7)	0.661	0.56 (−3.6 to 4.7)	0.778
Raster density										
Level 2	−0.16 (−2.1 to 1.7)	0.871	−0.39 (−2.2 to 2.9)	0.764	−0.49 (−3.3 to 2.3)	0.730	−0.26 (−2.9 to 3.4)	0.875	−0.41 (−2.8 to 1.9)	0.739
Level 3	−0.28 (−2.4 to 2.9)	0.836	−1.0 (−2.8 to 4.8)	0.600	−0.69 (−4.4 to 2.0)	0.713	−0.06 (−4.5 to 4.4)	0.980	−0.74 (−2.6 to 4.1)	0.665
Level 4	−1.1 (−6.4 to 4.2)	0.682	−2.0 (−8.8 to 4.7)	0.556	0.9 (−7.6 to 9.5)	0.824	0.97 (−9.7 to 7.8)	0.827	−0.23 (−6.9 to 6.4)	0.947

Reference levels were > 100 m for ≤ 100 m; > 200 m for ≤ 200 m and the 3-tiered gradient; 0 (roadways) for number of roadways ≤ 200 m of a residence; and level 1 for VMT/mi^2^ and raster density.

a All pulse pressure models were adjusted for age, sex, BMI, waist circumference, glucose level, HDL, LDL, smoking status, diabetes, prior heart attack, heart disease, statin use, hypertension medication, WBC count, income/poverty ratio, and education.
